# Rescue of Retinal Function by BDNF in a Mouse Model of Glaucoma

**DOI:** 10.1371/journal.pone.0115579

**Published:** 2014-12-23

**Authors:** Luciano Domenici, Nicola Origlia, Benedetto Falsini, Elisa Cerri, Davide Barloscio, Carlotta Fabiani, Marco Sansò, Luca Giovannini

**Affiliations:** 1 Neuroscience Institute of the National Council of Research (CNR), Pisa, Italy; 2 Department of Applied Clinical Sciences and Biotechnology (DISCAB), University of L’Aquila, L’Aquila, Italy; 3 Institute of Ophthalmology, Policlinico Gemelli, Catholic University, Rome, Italy; 4 Department of Translational Research on New Technologies in Medicine and Surgery, University of Pisa, Pisa, Italy; Dalhousie University, Canada

## Abstract

Vision loss in glaucoma is caused by progressive dysfunction of retinal ganglion cells (RGCs) and optic nerve atrophy. Here, we investigated the effectiveness of BDNF treatment to preserve vision in a glaucoma experimental model. As an established experimental model, we used the DBA/2J mouse, which develops chronic intraocular pressure (IOP) elevation that mimics primary open-angle glaucoma (POAG). IOP was measured at different ages in DBA/2J mice. Visual function was monitored using the steady-state Pattern Electroretinogram (P-ERG) and visual cortical evoked potentials (VEP). RGC alterations were assessed using Brn3 immunolabeling, and confocal microscope analysis. Human recombinant BDNF was dissolved in physiological solution (0.9% NaCl); the effects of repeated intravitreal injections and topical eye BDNF applications were independently evaluated in DBA/2J mice with ocular hypertension. BDNF level was measured in retinal homogenate by ELISA and western blot. We found a progressive decline of P-ERG and VEP responses in DBA/2J mice between 4 and 7 months of age, in relationship with the development of ocular hypertension and the reduction of Brn3 immunopositive RGCs. Conversely, repeated intravitreal injections (BDNF concentration = 2 µg/µl, volume = 1 µl, for each injection; 1 injection every four days, three injections over two weeks) and topical eye application of BDNF eye-drops (12 µg/µl, 5 µl eye-drop every 48 h for two weeks) were able to rescue visual responses in 7 month DBA/2J mice. In particular, BDNF topical eye treatment recovered P-ERG and VEP impairment increasing the number of Brn3 immunopositive RGCs. We showed that BDNF effects were independent of IOP reduction. Thus, topical eye treatment with BDNF represents a promisingly safe and feasible strategy to preserve visual function and diminish RGC vulnerability to ocular hypertension.

## Introduction

Glaucoma, a group of diseases characterized by progressive degeneration of the retinal ganglion cells (RGCs) and optic nerve fibers, is one of the leading causes of vision loss. A generally accepted theory suggests an initial insult to the axons of RGCs in the optic nerve head region, where they exit the eye [1]. Ocular hypertension is a risk factor frequently associated with the primary open-angle glaucoma (POAG), which accounts for half of the cases; indeed, drug treatments of glaucoma are essentially focused on lowering the intraocular pressure (IOP). However, despite the efficacy of therapies in reducing IOP, progressive vision impairment among glaucoma patients is common [2]. Thus, therapeutic approaches complementing IOP control are needed. We used the null mutant mouse DBA/2J as murine model of spontaneously arising glaucoma. DBA/2J mice are characterized by anterior chamber pathology consisting of pigment dispersion and development of iris stroma atrophy leading to blockade of vitreous humor drainage and progressive elevation of IOP [3]. In DBA/2J mice, elevated IOP is initially detected around 6 months of age, followed at later age by optic nerve atrophy, optic nerve head cupping and ganglion cell death [4]. In the present paper, we focus on DBA/2J mice at 6–7 months of age characterized by ocular hypertension and absence of RGC cell death [5]. Retinal visual function was assessed by the steady-state Pattern Electroretinogram (P-ERG). P-ERG is considered a reliable electrophysiological tool to investigate the functional state of the inner retina in humans and experimental animal models [6], [7], [8], [9], [10]; P-ERG generators were found in the inner retina [11], [12]. P-ERG was shown to be successful in detecting early visual impairment in glaucoma [13]. In our study, the P-ERG was related to the status of ganglion cells using markers such as transcription factor *Brn3*. The role of Brn3 factors in the development, differentiation, morphology and function of RGCs has been thoroughly studied in mice [14], [15]. The aim was to investigate potential alterations of RGCs in relationship with P-ERG impairment during an early stage of neurodegeneration characterized by ocular hypertension and absence of RGC cell death [5], [16], [17] in DBA/2J mice.

Visual Evoked Potentials (VEPs) were used to evaluate cortical responses and acuity (cortical spatial resolution limit).

Molecules with neurotrophic actions, including neurotrophic factors of NGF family (neurotrophins) such as the Brain Derived Neurotrophic Factor (BDNF), were shown to protect retinal cells in various models of optic nerve injury and disease [18], [19], [20]. BDNF is a protein locally produced by cells in the ganglion cell and inner nuclear layers [21]; its TrkB receptor is expressed in RGCs, amacrine and Müller cells [22] that represent the cellular target of BDNF trophic action [23], [24], [25]. Interestingly, supplying BDNF by intravitreal injection results in a partial protective effect in animal model of glaucoma [26].

So far, BDNF was typically administered to the internal ocular tissues by intravitreal injection; this method of treatment is associated with the risk of various complications such as ocular bulb perforation and infections. In addition, recent studies suggested that intravitreal injections (or eye cannulation) compromise the immune privilege of the eye and activates immunological responses [27].

We separately used repeated intravitreal and topical eye treatments with the aim of exploiting the possibility of administering BDNF using the external ophthalmic route. Treatments with BDNF were carried out in 7 month DBA/2J mice with ocular hypertension. We found that BDNF topical eye treatment prevented visual impairment in the DBA/2J mouse without interfering with IOP.

## Materials and Methods

### Ethics statement

All experiments were conducted according to Ministry of Health (the regulatory authority for controlling the use of laboratory animals and ethics on animal experiments in Italy) guidelines (Legislative Decree n. 116/92) and in accordance with the European Community guidelines (European Directive 86/609/EEC). The experimental protocol (IACUC document) was approved by Ministry of Health (n. 152/2013; IACUC is renewed every three years). Local branch of the Ministry of Health in Pisa controls the correct application of the approved experimental protocols. One veterinary is specifically trained in experimental protocols involving animal use, care and safety.

### Animals and BDNF treatment

Animals were adult Wistar rats, adult C57BL/6J (5–7 months of age) and DBA/2J mice of different ages (Charles River Laboratories International Inc., Santangelo Lodigiano, Italy) reared on a 12-h light/dark cycle (animals were kept in a 12 h light/12 h dark cycle, with the illumination level below 60 photopic lux). Repeated measures of IOP were performed using TonoLab tonometer (Icare, Finland) in unanaesthetized mice trained to take position in a partial restrainer without invasive handling; the tonometer was held and maneuvered through the z-axis of a micromanipulator, IOP was measured in the morning 10–12 am to avoid oscillation. We collected at least 3 to 4 measurements from each eye; treated eyes were evaluated before, during and after the end of treatment.

Human recombinant BDNF (QED Bioscience Inc., San Diego, USA) was dissolved in physiological solution (0.9% NaCl) to obtain the concentrations of 1, 2, 5 and 12 µg/µl with the final pH of 6.7–7; particular care was dedicated to maintain pH and osmolarity of the BDNF solution in the range of physiological values. For topical eye treatment, mice and rats received a drop (5 and 10 µl, respectively) of BDNF solution at the level of conjunctival fornix. Contralateral eyes were treated with an equal volume of vehicle (0.9% NaCl); in the mouse, 5 µl volume was administered in two times due to small size of the eye. BDNF solution was also intravitreally injected in a few rats and mice anaesthetized with Avertin (2 ml/100 g.b.w.); 2 µl (1 µg/µl of BDNF) was injected into one rat eye and 1 µl (2 µg/µl of BDNF) into a mouse eye through a pulled glass capillary inserted just behind the corneo-scleral margin using a micro-syringe injector (Sutter Instruments Co., USA). The contralateral eye was intravitreally injected with the same volume of vehicle. In a few mice we used denatured BDNF (95°C during 10 min) to control potential side-effects of BDNF eye treatment.

After the eye treatments both eyes were protected using TSP solution (artificial tears, 0.5% solution, Farmigea Holding, Pisa, Italy).

### Electroretinograms and visual evoked potentials

Mice were anaesthetized by an intraperitoneal injection of urethane (20% sol., 0.1 ml/100 g. b.w.; Sigma-Aldrich, USA) for long recording sessions or Avertin (2 ml/100 g.b.w.) for short recording sessions (the effect of this anaesthetic lasted no more than one-two hours) and then were mounted in a stereotaxic apparatus allowing a full view of the visual stimulus (monitor Sony; average mean luminance = 20 cd/m^2^) and left to adapt for half-hour before starting electrophysiological recording. Body temperature was maintained around 38°C and the heart monitored. Following the illustrated experimental protocol, the fissured pupil was maintained for several hours without the development of cataracts in a large number of mice; the pupil direction (the pupil was projected on a tangential white blackboard before, during and at the end of the recording session) and size was checked through the recording session. The few mice, which developed cataracts were excluded.

Visual stimuli consisted of square wave horizontal gratings of various spatial frequencies (0.05–0.5 c/deg) and high contrast (90%); gratings were computer generated (Cambridge Research Systems, UK, VSG 2.2 card) and sinusoidally alternated in counterphase at 4 Hz (steady-state P-ERG).

Spatial frequencies of less than 0.05 cycle/deg were not used because it was technically impossible to include a sufficient number of cycles (at least three) on our computer screen. Corneal electrodes were connected through isolated wires to filters and amplifiers (Digitimer, Neurolog, UK); the signals were amplified 20000-fold, band-pass filtered (0.3–100 Hz), digitized, and averaged. The relative power for each harmonic was given as the percentage of total spectral power using Fast Fourier Transform [7]. To increase the signal to noise ratio at least 200 responses in blocks of ten events each were averaged looking at the signal to noise ratio; the amplitude and phase of the first two harmonics were available on-line and the signal was stored for further off-line analysis. We analyzed the amplitude of the P-ERG signal and second harmonic (signals with a second harmonic lower than 50% of the total power measured by Fast Fourier Transform were excluded) as a function of spatial frequency. The noise level for each spatial frequency (contrast = 0) was also measured.

Cone-driven responses were evoked by photopic flash (F-ERG; flash intensity = 20–25 cd/m^2^×s^−1^, background light = 15–25 cd/m^2^, interstimulus interval = 5 s); for this purpose the mouse was placed in a Ganzfeld stimulator (LACE, Italy). Ten responses were averaged and the b-wave response analyzed.

Visual evoked potentials (VEPs) were recorded through a screw used for in vivo continuous EEG recordings (stainless steel, 1 mm in diameter; FHC Inc., USA) placed on top of the cranial occipital bone, in a superficial hole of the occipital bone made by a cranial drill in the correspondence of area Oc1b (localized in a stereotaxix apparatus; preliminary recordings sessions were dedicated to finding the correspondence between the screw position and area Oc1b, using recording microelectrodes inserted in the Oc1b; [28]) contralateral to the stimulated eye; the screw was positioned and fixed using dentist cement on top of the cranial bone and connected through an isolated wire to filters and amplifiers (Digitimer, Neurolog). The electrical signals were amplified (10000-fold), band-pass filtered (0.3–100 Hz), digitized, and averaged (at least 100 responses in blocks of ten events each). The VEPs in response to an abrupt contrast reversal (1 Hz) were evaluated in the time domain by measuring the peak-to-trough amplitude and peak latency of the major component (transient VEP, P100). The VEP amplitudes evoked by alternating gratings were analyzed as a function of spatial frequency.

### Retinal immunocytochemistry

At the end of the recording sessions, mice were sacrificed after injection of additional supply of urethane and their eyecups were harvested. After removal of the anterior segment the posterior portion of the eye was fixed by immersion in a solution of 4% paraformaldehyde (PFA) in 0.01 phosphate buffer (PB). Four cuts were then made to flatten the tissue. This procedure resulted in retinas with 4 lobes corresponding to the superior, temporal, inferior, and nasal lobe. Flat whole mounted retinas from treated, contralateral vehicle treated and untreated eyes were prepared and processed. Briefly, retinas were incubated for 5 days with the primary antibody against Brn3 (Santa-Cruz, Ca-USA) in PB (0.1 M+1% BSA and 0.1% Triton X100; antibody dilution = 1/200) or γ-Synuclein (Abcam, USA; antibody dilution = 1/200) followed by several washes (10 min/each) in PB and incubated with secondary antibody FITC labeled (Alexa 488, concentration = 1/500) for 2 days. For quantification of immunopositive RGCs, each retinal quadrant was further subdivided in regions of acquisition defined as peripheral retina (the retinal portion roughly 2.5 mm from the optic nerve head) and as central retina (the retinal portion in the nearby area, 1 mm, of the optic nerve head). Our sampling method was adapted from previous retinal protocols [29] according to the estimated density of the RGCs in pigmented mice. Series were obtained in at least four different windows for each retinal quadrant in two different retinal sites, peripheral and central regions. In this way labeled cells were identified, counted and pooled together from 32 fields (250×250 µm) spaced along the dorsal–ventral and nasal–temporal retinal meridians for each retina maintaining separate the central and peripheral regions. For each field, serial optical sections were acquired using a Leica TCS-NT confocal microscope equipped with a krypton-argon laser and high resolution images (1024×1024 pixels) were obtained using Plan-Apocromatic 40/1.2 objective. The optical sections were 1 µm apart. For each series, 3 µM Z-stack of images were acquired within the ganglion cell layer.

### ELISA

Rats and mice were deeply anaesthetized with an intraperitoneal urethane injection and sacrificed at different time intervals following BDNF treatment. The eyes were then removed, and the BDNF level measured in the retinal homogenate and vitreous humor of both the eye treated with BDNF and the other eye treated with the carrier solution. Measurements were conducted by immunoassay (ELISA; BDNF Emax immunoassay system, Promega, Madison, WI, USA) following standard protocols. Briefly, quantification of BDNF was detected in protein extracts from the retina and fixed volume of the vitreous sample. Cell pellets were collected, immediately frozen at −80°C. Cell pellets were subsequently lysed in cell extraction buffer (10 mM Tris, pH 7.4, 100 mM NaCl, 1 mM EDTA, 1 mM EGTA, 1 mM NaF, 20 mM Na4P2O7, 2 mM Na3VO4, 1% Triton X-100, 10% glycerol, 0.1% SDS, 0.5% deoxycholate, and 1 mM PMSF; Sigma protease mixture inhibitor). The extract was then centrifuged (13000 rpm, 10 min at 4°C) to obtain a clear lysate that was used for the assay. Retinal samples were pipetted into wells coated with a specific monoclonal antibody against BDNF; after washing, the detection antibody (rabbit anti-BDNF) was added to wells. For measurement of BDNF in the vitreous, 1 µl was added to 9 µl of sample buffer and added to ELISA plates for analysis. The sandwich was completed by adding anti-rabbit IgG-HRP conjugated. After completing the reaction with stabilized chromogen (tetramethylbenzidine), absorbance was read at 450 nm using a microplate reader (Bio-Rad, USA). Values were expressed as pg/mg of protein for the retina (values were normalized in respect to the total protein content determined with a colorimetric assay, Bio-Rad).

### Western Blot

Retinas of different mice were dissected from eyes. The retinal homogenates from at least two retinas/each mouse group were processed using western blot. Briefly, an equal amount of proteins (50 µg) were resolved electrophoretically on a 12% SDS-PAGE Bis-TRIS gel. Proteins were transferred on a nitrocellulose membrane 0.45 µm, followed blocking in TBS 5% dry skimmed milk and 0.2% Tween-20 and incubation with two anti-BDNF different primary antibodies (AB1534SP, Chemicon/Millipore, USA, 1/500 and Santa Cruz N-20 SC546, 1/400 were separately used) at 4°C overnight in TBS with 0.1% Tween-20. After washing the membranes were incubated with anti-rabbit IgG antibody (1/20000, Cell Signaling, USA), HRP-linked for 1 h at room temperature in TBS with 0.2% Tween-20. As control in a dedicated lane, we used a known amount of human recombinant BDNF. β-tubulin (1/2000 in BSA 3%, Cell Signaling) was used as a house-keeping protein. Proteins were detected through an enhanced chemiluminescent (ECL) detection system (Clarity Western ECL Substrate, Bio-Rad). Images were acquired with Molecular Imager ChemiDoc XRS + System (Bio-Rad) and data were analyzed using Quantity One Software (Bio-Rad).

### Animal groups

We investigated C57BL/6J adult mice (5–7 months of age), DBA/2J mice (4, 6, 7, 8 months of age).

### Statistics

The Student’s *t* test and one-way ANOVA (SigmaStat, Jandell Scientific, USA) followed by pairwise multiple comparisons test (Student-Newman-Keuls pairwise multiple comparisons unless otherwise stated) were used for statistical analysis. Regression function and correlation (Pearson Product Moment Correlation) tests were also used. A p value<0.05 was considered statistically significant. Histograms were plotted using single case distribution, mean values ± SEM or SD as appropriate.

## Results

### P-ERG and VEP are impaired in DBA/2J murine model of glaucoma

**Figure 1:**
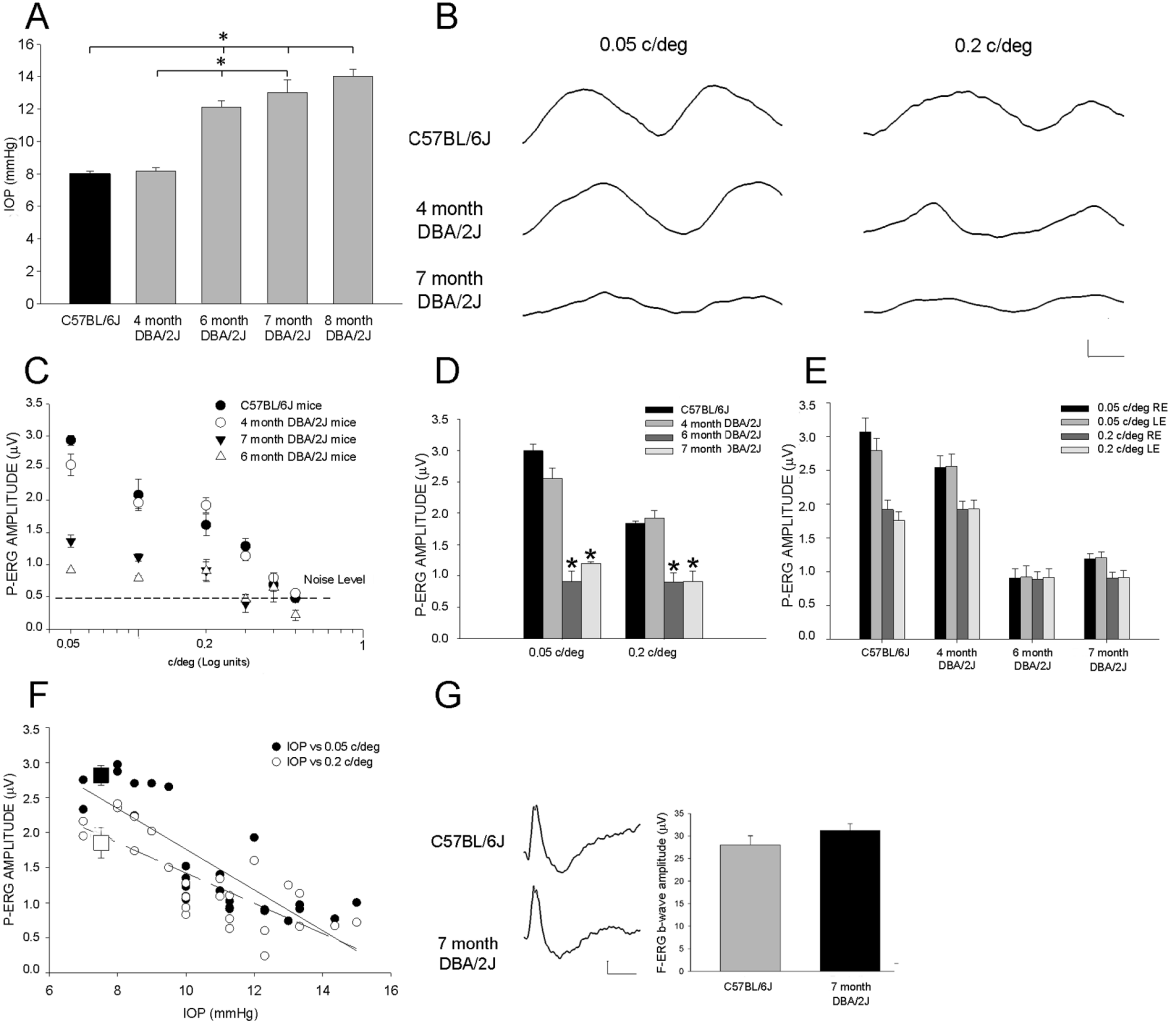
IOP and P-ERG in DBA/2J mice. **A**. IOP recorded by TonoLab was plotted as mean value (mmHg) in C57BL/6J, 4 month, 6 month, 7 month and 8 month DBA/2J mice; 6, 7 and 8 month DBA/2J *versus* C57BL/6J and 4 month DBA/2J mice, *p<0.05, one-way ANOVA. **B**. Examples of P-ERG recordings from C57BL/6J (1^st^ row), 4 month (2^nd^ row) and 7 month (3^rd^ bottom row) DBA/2J mice; P-ERG (4 Hz frequency of alternation) was recorded at 0.05 (left column) and 0.2 c/deg (right column), 90% contrast. The steady-state P-ERG evoked by frequency of alternation of 4 Hz assumes a clear sinusoidal profile centered on the second harmonic (8 Hz); the second harmonic is the most represented (relative power higher than 50% as assessed using Fast Fourier Transform, see material and methods). Horizontal calibration bar = 50 msec, vertical calibration bar = 1 µV. **C**. The averaged amplitudes of P-ERG in response to gratings of different spatial frequency were plotted as a function of spatial frequency, c/deg (semi-logarithmic scale); the mean noise level (responses recorded at different spatial frequencies with contrast = 0) is visible as a dotted line. Mean P-ERG amplitude was reduced at all spatial frequencies in 6 and 7 month DBA/2J mice. **D**. The histogram reports the P-ERG amplitudes at 0.05 and 0.2 c/deg in C57BL/6J, 4, 6 and 7 month DBA/2J mice; error bars indicate SEM; *p<0.05 (6 and 7 month DBA/2J mice *versus* 4 month DBA/2J and C57BL/6J mice). **E**. P-ERG amplitudes at 0.05 and 0.2 c/deg of single eyes (right and left eyes, RE and LE, respectively) were plotted to show interocular symmetry of P-ERG responses. **F**. Scatter plot of PERG amplitudes vs. IOP in 4, 6 and 7 month DBA/2J mice (for spatial frequencies of 0.05 and 0.2 c/deg). At both frequencies P-ERG amplitudes were inversely correlated to IOP. The solid line is the best linear fit for the 0.05 c/deg; the black square indicates the mean P-ERG amplitude ± SEM in C57BL/6J mice. The dashed line is the best linear fit for 0.2 c/deg; the empty square indicates the mean P-ERG amplitude ± SEM in C57BL/6J mice. **G**. The left panel displays examples of F-ERG evoked by a flash of 25 cd/m^2^×s^−1^ intensity in the presence of constant background of 15 cd/m^2^: top row reports an example from C57BL/6J mouse, bottom row shows an example from 7 month DBA/2J mouse. Horizontal calibration bar = 100 ms, vertical calibration bar = 5 µV. The right panel shows the averaged b-wave amplitude in C57BL/6J (n mice = 4, 8 eyes; mean = 28.02 µV, SEM = 0.2) and 7 month DBA/2J mice (n mice = 4, 8 eyes; mean = 31.23 µV, SEM = 1.47).

As a model of spontaneously arising glaucoma we used the DBA/2J mouse, which develops ocular hypertension. IOP measured by TonoLab was already elevated in 6 month-old-DBA/2J mice (C57BL/6J, n = 12 eyes, mean = 8 mmHg, SEM = 0.17; 4 month DBA/2J, n eyes = 10, mean = 8.75, SEM = 0.47; 6 month DBA/2J, n eyes = 8, mean = 12.44, SEM = 0.41) as shown in Fig. 1 (A). IOP elevation persists at 7 and 8 months of age (Fig. 1A) and at later ages. In the same strain of DBA/2J mice IOP was in the normal range when measured in younger animals, at the age of 4 months (Fig. 1A).

Inner retinal function was evaluated by P-ERG recording. We used the steady-state P-ERG evoked at 4 Hz frequency of alternation, high contrast (90%). In Fig. 1 (B), we reported examples of P-ERG response traces evoked by two different spatial frequencies, 0.05 and 0.2 c/deg; P-ERG waveforms assume a clear sinusoidal profile. In Fig. 1 (C) we plotted the P-ERG amplitude at different spatial frequencies (0.05–0.5 c/deg) in adult C57BL/6J mice, adult DBA/2J of 4, 6 and 7 months of age. Fig. 1 (C) shows that the mean P-ERG amplitude in C57BL/6J progressively decreases as the spatial frequency (x-axis, Log units) increases becoming almost indistinguishable from noise at spatial frequencies higher than 0.5 c/deg, i.e. when the retinal spatial resolution limit is approached [28]. The same curve is obtained in 4 month DBA/2J mice with normal IOP. On the contrary, the P-ERG amplitude at all tested spatial frequencies was strongly reduced in 6 and 7 month DBA/2J mice characterized by ocular hypertension; only spatial frequencies comprised between 0.05 and 0.2 c/deg resulted reliably over the mean noise level in DBA/2J mice at this age. For this reason, we focused on P-ERG recordings in response to visual stimuli of 0.05 and 0.2 c/deg. Fig. 1 (D) shows that the amplitudes of P-ERG evoked by 0.05 and 0.2 c/deg spatial frequency were significantly reduced in 6 and 7 month DBA/2J mice (6 months of age, n. mice = 8, eyes = 16; 0.05 c/deg, mean = 0.91 µV, SEM = 0.02 and 0.2 c/deg, mean = 0.9 µV, SEM = 0.1; 7 months of age, n mice = 8, eyes = 16; 0.05 c/deg mean = 1.12 µV, SEM = 0.07 and 0.2 c/deg mean = 0.91 µV, SEM = 0.07) with respect to age matched C57BL/6J (n mice = 8, eyes = 16, 0.05 c/deg mean = 2.9 µV, SEM = 0.08 and 0.2 c/deg mean = 1.84 µV, SEM = 0.04) and 4 month DBA/2J mouse with normal IOP (n mice = 10, eyes = 20; 0.05 c/deg mean = 2.58 µV, SEM = 0.1 and 0.2 c/deg, mean = 1.92 µV, SEM = 0.1). In Fig. 1 (E) right and left eyes were plotted separately to show the interocular variability (ocular symmetry) of P-ERG recorded at 0.05 and 0.2 c/deg. We also found that P-ERG amplitudes at spatial frequencies of 0.05 and 0.2 c/deg were inversely related to IOP (Fig. 1F) in DBA/2J mice (Pearson’s correlation coefficient r = −0.8, p = 0.00000027 for 0.05c/deg; r = −0.77, p = 0.00000187 for 0.2 c/deg); we also used multiple linear regression statistics with age and IOP as independent variable. We found that IOP and age contribute to predicting 0.05 and 0.2 c/deg amplitude reduction. These results underline the importance of ocular hypertension during a period comprised between 4 and 7 months of age in the DBA/2J mice.

To gain information on the physiology of the outer retina we investigated the cone-driven response to flash ERG in photopic condition; we did not observe gross alteration of photopic F-ERG in 7 month DBA/2J mice (Fig. 1G).

**Figure 2:**
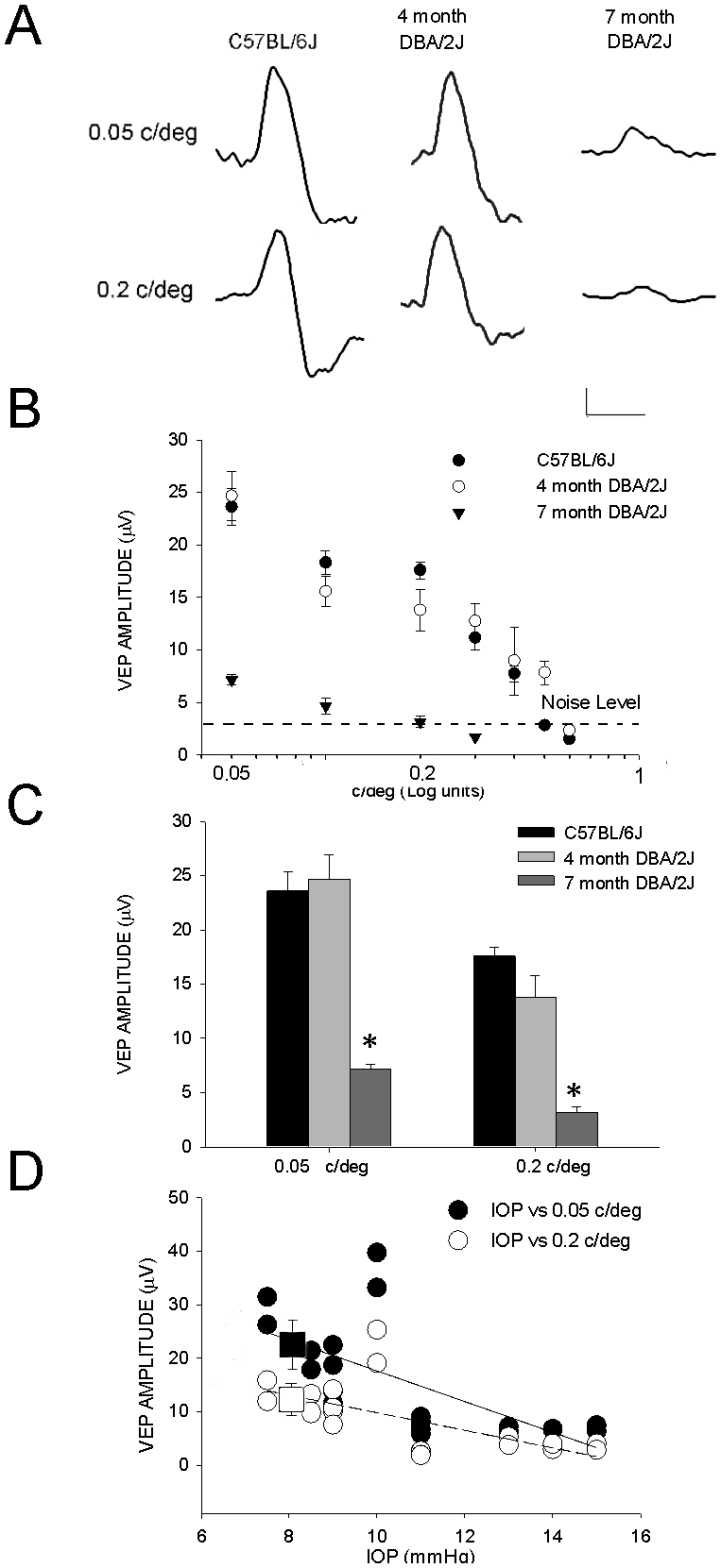
VEP in DBA/2J mice. **A**. Examples of VEP recordings from C57BL/6J (1^st^ column), 4 month (middle column) and 7 month DBA/2J mice (3^rd^ lateral column); VEPs were recorded through a screw inserted in the occipital bone contralateral to the stimulated eye in response to stimuli of 0.05 (1^st^ row) and 0.2 c/deg (2^nd^ row), 90% contrast (1 Hz frequency of alternation). VEPs show a prominent positive component characterized by latency around 107 msec in C57BL/6J mouse. Horizontal calibration bar = 100 msec, vertical calibration bar = 5 µV. **B**. The averaged amplitudes of VEP in C57BL/6J, 4 month and 7 month DBA/2J mice were plotted as a function of the spatial frequencies (semi-logarithmic scale); the mean noise level (responses recorded at different spatial frequencies with contrast = 0) is visible as a dotted line. Mean VEP amplitudes were reduced at all spatial frequencies in 7 month DBA/2J mice with respect to 4 month DBA/2J and C57BL/6J mice. **C**. Mean VEP amplitudes at 0.05 and 0.2 c/deg in C57BL/6J, 4 month and 7 month DBA/2J mice; 7 month DBA/2J *versus* C57BL/6J and 4 month DBA/2J, *p<0.05 (one-way ANOVA). Error bars indicate SEM. **D**. Scatter plot of VEP amplitudes vs. IOP in 4 month and 7 month DBA/2J mice (for spatial frequencies of 0.05 and 0.2 c/deg). At both frequencies VEP amplitudes were inversely correlated to IOP in DBA/2J mice. The dashed line is the best linear fit for 0.2 c/deg. The solid line is the best linear fit for the 0.05 c/deg. The black square indicates the mean VEP amplitude ± SEM at 0.05 c/deg in C57BL/6J mice and the empty square indicates the mean VEP amplitude ± SEM at 0.2 c/deg in C57BL/6J mice.

To further explore the visual function of 4 and 7 month DBA/2J mice, we measured the amplitude of VEPs evoked by alternating gratings. VEPs elicited by pattern alternating at 1 Hz were automatically analyzed as transient responses to measure the amplitudes as a function of different spatial frequencies. At the different spatial frequencies tested (0.05–0.5 cycle/deg, contrast 90%), VEPs recorded through a screw placed on top of cranial occipital bone contralateral to the stimulated eye in C57BL7/6J mice consisted of a major positive component with a mean latency of 107 msec (SEM = 0.003); examples of VEPs recorded in C57BL/6J, 4 month and 7 month DBA/2J mice are shown in Fig. 2A. In the C57BL/6J mice and 4 month DBA/2J mice with normal IOP, VEP amplitudes (Fig. 2B) decreased from 0.05 to 0.5 cycle/deg until the signal became indistinguishable from the noise level. Similarly to P-ERG, the mean VEP amplitudes at all tested spatial frequencies were strongly reduced in 7 month DBA/2J mice with respect to age matched C57BL/6J and 4 month DBA/2J mice. From the Fig. 2 (C) it is clear that the VEP amplitudes at 0.05 and 0.2 c/deg were drastically reduced in 7 month DBA/2J mice (n mice = 5, eyes = 10; 0.05 c/deg, mean = 7.05 µV, SEM = 0.38 and 0.2 c/deg, mean = 3.22 µV, SEM = 0.41) with respect to C57BL/6J mice (n mice = 6, eyes = 12; 0.05 c/deg, mean = 23.6 µV, SEM = 1.78 and 0.2 c/deg, mean = 17.58 µV, SEM = 0.81) and 4 month DBA/2J mice (n mice = 5, eyes = 10, 0.05 c/deg, mean = 23.68 µV, SEM = 4 and 0.2 c/deg, mean = 13.8 µV, SEM = 2.25). In panel D (Fig. 2) we showed that the VEP amplitudes at spatial frequencies of 0.05 and 0.2 c/deg were inversely related to IOP in DBA/2J mice (Pearson’s correlation coefficient r = − 0.662, p = 0.0015 for 0.05c/deg; r = − 0.61, p = 0.0043 for 0.2 c/deg), similarly to that shown for P-ERG.

Thus, visual response as assessed by P-ERG and VEP resulted impaired in DBA/2J mice with ocular hypertension. Hereinafter, we focused on 7 month DBA/2J mice characterized by ocular hypertension and impairment of P-ERG and VEP.

### Brn3 labelled retinal cells are decreased in the RGC layer of DBA/2J mice

At the end of P-ERG recording sessions retinal tissues were processed for immunocytochemistry.

**Figure 3:**
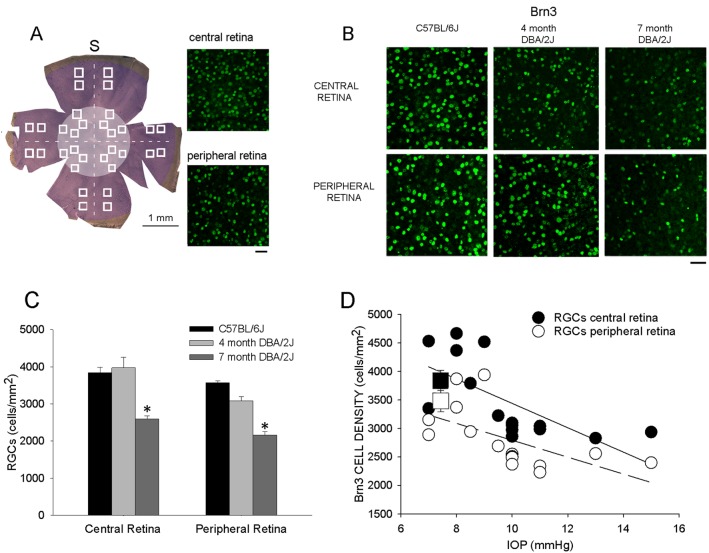
RGCs expressing Brn3 in DBA/2J mice. **A**. On the left, low power view of a flat whole mount retina is shown. Different retinal fields (white border squares) in the central (light brown disk) and peripheral retina (dark brown) were acquired along the horizontal (temporo-nasal) and vertical (superior-inferior pole) axis centered on the optic nerve head; T as temporal and S as superior. For each retinal region (central and peripheral retina) of the four sectors, 4 retinal fields were selected. Brn3 immunopositive RGCs were counted in each field and pooled together maintaining separate peripheral and central regions. An example of Brn3 expression (green) in RGCs of a C57BL/6J retina is visible on the right; top and bottom microphotographies correspond to the central and peripheral retina, respectively. Images are projections of different Z-stacks of the ganglion cell layer on a single plane. All immunopositive cells were counted independently from the level of Brn3 expression; scale bar = 40 µm. **B**. Images from central (1^st^ row) and peripheral (2^nd^ row) retina showing Brn3 immunopositive RGCs in C57BL/6J mouse (1^st^ column), 4 month DBA/2J (middle column) and in 7 month DBA/2J mouse (right column); scale bar = 40 µm. **C**. Mean cell density of RGCs expressing Brn3 (cells/mm^2^) in central and peripheral retina from flat whole mounted retinas of C57BL/6J, 4 month and 7 month DBA/2J mice; Brn3 RGCs in 7 month DBA/2J *versus* Brn3 RGCs in C57BL/6J and 4 month DBA/2J mice, *p<0.05 (one-way ANOVA). **D**. Scatter plot of RGCs expressing Brn3 vs. IOP in 4 month and 7 month DBA/2J mice, for central and peripheral retina. The solid line is the best linear fit for RGCs expressing Brn3 in the central retina. The dashed line is the best linear fit for RGCs expressing Brn3 in the peripheral retina; dark square ± SEM and empty square ± SEM represent mean RGCs in central and peripheral retina, respectively, of C57BL/6J mice. Brn3 cell density in central and peripheral retina of DBA/2J mice resulted inversely correlated with IOP.

Characterizing markers for RGC impairment could be an important step in understanding the molecular and cellular processes in DBA/2J mice with ocular hypertension. Using Brn3 antibody recognizing the three products of Brn3 isoforms (a, b, c) and confocal microscopy analysis, we conducted quantification of Brn3 immunopositive cell density in the RGC layer of whole mount retinas. Fig. 3 (A) shows a low magnification image of a flat mounted retina divided in 4 quadrants disposed along the temporo-nasal and vertical axis centered on the optic nerve head. At higher magnification on the right, Brn3 labeled cells are visible (green) in the central and peripheral retina of a C57BL/6J mouse; retinal cells were sampled in peripheral and central retina of the 4 quadrants (32 fields from each retina were sampled, see material and methods). Brn3 labeled RGCs (Fig. 3B) appear reduced in number both in the central and peripheral retina of 7 month DBA/2J mice in respect to C57BL/6J mice and 4 month DBA/2J mice.

The histogram reported in Fig. 3 (C) shows a significant higher cell density of Brn3 labeled RGCs in C57BL/6J and young 4 month DBA/2J with normal IOP with respect to 7 month DBA/2J with ocular hypertension (C57BL/6J, n = 5 mice, eyes = 10, central retina mean cell density = 3845, SEM = 137 and peripheral retina mean cell density = 3569, SEM = 83; 4 month DBA/2J mice, n = 7 mice, eyes = 14, central retina mean cell density = 3971, SEM = 306 and peripheral retina mean cell density = 3084, SEM = 305; 7 month DBA/2J mice, n mice = 9, eyes = 18 central retina mean cell density = 2592.77, SEM = 38.24 and peripheral retina mean cell density = 2159, SEM = 41.37; p<0.05).

Reduction of Brn3 immunopositive RGCs was related to IOP in DBA/2J mice of different ages; as the IOP increases Brn3 RGCs decrease as shown in Fig. 3 (D). Thus, expression of Brn3 in RGCs was altered in temporal relationship with IOP rise.

In S1 Fig., we reported additional data showing that RGCs immunolabeled by a second marker, namely γ-Synuclein, a member of small unfolded proteins (Synucleins) were not reduced in 7 month DBA/2J mice starting to be reduced at 11–14 months of age. In addition, we did not find apoptotic RGCs in the retina of DBA/2J mice with ocular hypertension during the period comprised between 7 and 8 months (Tunel method in whole mount retina, negative data not shown).

### BDNF topical eye treatment preserves P-ERG and VEP responses in DBA/2J mice

**Figure 4:**
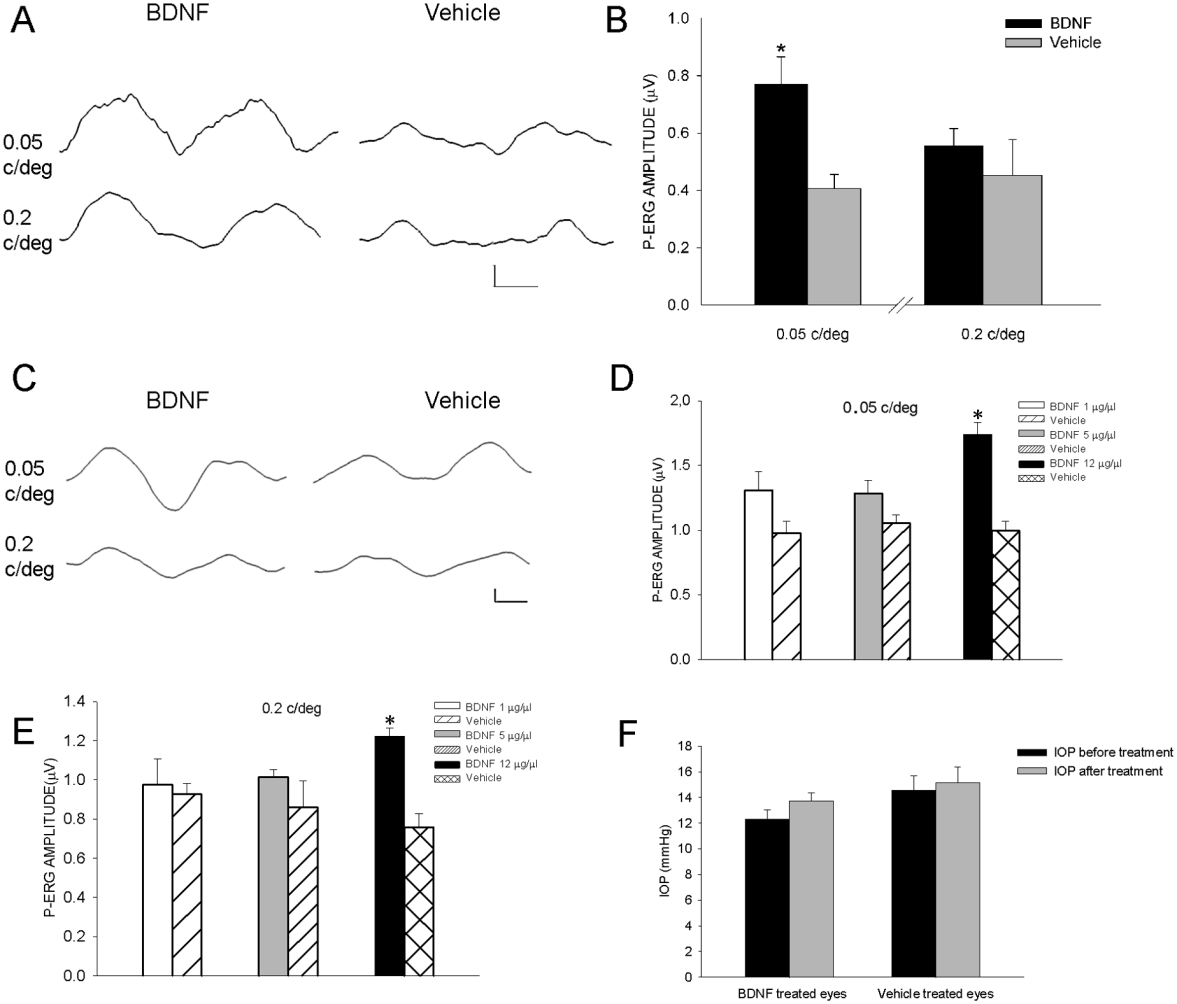
Effects of eye treatments with BDNF on P-ERG of DBA/2J mice. **A, B**. Intravitreal treatment consisted of three intravitral injections over two weeks (BDNF = 2 µg/µl, volume = 1 µl for each injection) in 7 month DBA/2J mice (n = 5, n eyes = 5) with ocular hypertension; the contralateral eye was injected with the physiological solution (vehicle). **A**. Examples of P-ERG recorded from BDNF injected eye (1^st^ column) and contralateral vehicle treated eye (2^nd^ column); P-ERG was evoked by 0.05 and 0.2 c/deg spatial frequencies, 1^st^ and 2^nd^ row, respectively. Horizontal calibration bar = 50 msec, vertical calibration bar = 1 µV. **B**. Mean P-ERG amplitude at spatial frequency of 0.05 c/deg and 0.2 c/deg for BDNF and vehicle injected eyes in 7 month DBA/2J mice; * p<0.05. **C–F**. Topical eye treatment consisted of 1 drop of 5 µl containing BDNF at different concentrations or vehicle (physiological solution) for two weeks, one application every 48 h, in 7 month DBA/2J mice with ocular hypertension. **C**. Examples of P-ERG recorded from BDNF treated eye (12 µg/µl, 1^st^ column) and contralateral vehicle treated eye (2^nd^ column); P-ERG was evoked by 0.05 and 0.2 c/deg spatial frequencies, 1^st^ and 2^nd^ row, respectively. Horizontal calibration bar = 50 msec, vertical calibration bar = 1 µV. **D** and **E**. Mean P-ERG amplitude at spatial frequency of 0.05 c/deg (D) and 0.2 c/deg (E) from the eyes of 7 month DBA/2J mice treated with BDNF at concentrations of 1, 5 and 12 µg/µl; the averaged P-ERG amplitude in the contralateral eye treated with vehicle is reported for statistical comparison; BDNF (12 µg/µl) *versus* vehicle treated eyes, 1 and 5 µg/µl BDNF, * p<0.05 (one-way ANOVA). **F**. IOP measured before and after treatment in BDNF treated eyes (12 µg/µl, n eyes = 7) and vehicle (n eyes = 7). Error bars indicate SEM.

Previous reports [30], [31] showed that intravitreal injection of BDNF at concentrations comprised between 1 and 10 µg/µl was able to protect retinal cells in different animal models of retinal degeneration. At first, we used repeated intravitreal injections of BDNF in a few 7 month DBA/2J mice (n mice = 5); intravitreal injection is the technique most commonly used for acute treatment in animal models of neurodegenerative disorders to allow a protein characterised by a high molecular weight and low propensity to pass through the various eye barriers to be carried to the retina. An example of P-ERG recorded from the eye treated for two weeks with three intravitreal injections of human recombinant BDNF (concentration = 2 µg/µl, volume = 1 µl, for each injection; 1 injection every four days, three injections over two weeks; total dose/each mouse eye = 6 µg) is shown in Fig. 4 (A). In Fig. 4B we showed that three repeated intravitreal injections of BDNF in a few 7 month DBA/2J mice (n mice = 5, 5 eyes) induced a rescue of P-ERG, especially at 0.05 c/deg. However, multiple intravitreal injections are unlikely to be a sustainable treatment for a mouse eye with ocular hypertension; indeed, in our experimental conditions, no more than three intravitreal injections over two weeks were tolerated in 7 month DBA/2J mice. This prompted us to explore an easy, safe and repeatable topical eye method of treatment, which is our main focus. To this aim we treated one eye of DBA/2J mice (7 months of age) with repeated topical eye applications of human recombinant BDNF (BDNF was diluted in 1 drop of 5 µl physiological solution; the topical treatment lasted two weeks, six applications, one application every 48 h); the contralateral eye received the application of the same volume of physiological solution during the same period. We used three different concentrations of human recombinant BDNF: 1, 5 and 12 µg/µl (30, 150 and 360 µg, respectively, total dose/each mouse eye). An example of P-ERG recorded from the eye treated for two weeks with six topical eye applications of BDNF is shown in Fig. 4 (C, BDNF concentration = 12 µg/µl). The results in Fig. 4 (D, E) show that the amplitudes of P-ERG recorded at spatial frequencies of 0.05 and 0.2 c/deg were significantly increased by 12 µg/µl BDNF (n mice = 9, eyes = 9; 0.05 c/deg mean amplitude = 1.8 µV, SEM = 0.07 and 0.2 c/deg mean amplitude = 1.22 µV, SEM = 0.04) with respect to the P-ERG of vehicle contralateral treated eye (n eyes = 9; 0.05 c/deg mean amplitude = 1.08 µV, SEM = 0.09 and 0.2 c/deg mean amplitude = 0.88 µV, SEM = 0.06); 1 and 5 µg/µl concentrations resulted unable to rescue the P-ERG in the BDNF treated eye. To further control for potential side-effects of BDNF treatment we used treatment of one eye with denatured BDNF in a few 7 month DBA/2J mice (n = 4; BDNF treated eye, P-ERG amplitude at 0.05 c/deg = 1.6 µV±0.2; denatured BDNF treated eye, P-ERG amplitude at 0.05 c/deg = 1 µV±0.1); thus the treatment with denatured BDNF was unable to rescue the P-ERG in DBA/2J mice.

In Fig. 4 (F) we reported that repeated topical applications of BDNF at the highest concentration did not influence IOP, thus excluding the possibility that P-ERG rescue by BDNF was due to IOP changes.

**Figure 5:**
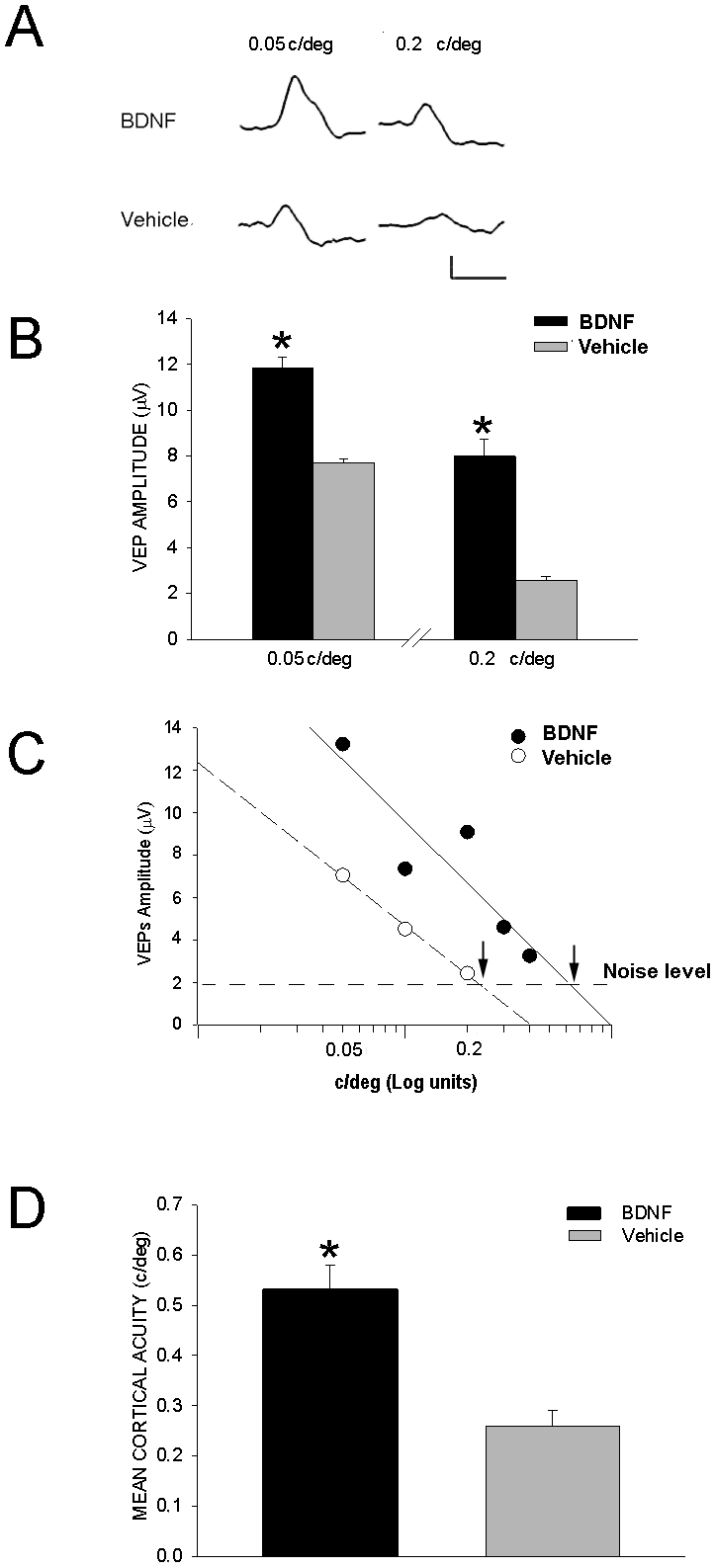
Effects of topical eye treatment with BDNF on VEP of DBA/2J mice. Topical eye treatment consisted of 1 drop of 5 µl containing BDNF (12 µg/µl) or physiological solution (vehicle) for two weeks, one application every 48 h, in 7 month DBA/2J mice with ocular hypertension. VEPs were recorded through a screw inserted in the occipital bone contralateral to the stimulated eye. **A**. Examples of VEPs recorded for stimulation of BDNF treated eye (top row) and contralateral vehicle treated eye (bottom row); VEP was evoked by 0.05 and 0.2 c/deg, 1^st^ and 2^nd^ column, respectively. Calibration horizontal bar = 100 msec, vertical bar = 1 µV. **B**. The histogram reports the mean VEP amplitudes at 0.05 and 0.2 c/deg in 12 µg/µl BDNF treated eyes and vehicle treated eyes of 7 month DBA/2J mice; BDNF treated eyes *versus* vehicle treated eyes, *p<0.05 (one-way ANOVA). **C**. The cortical spatial resolution limit was calculated by linear extrapolation to noise level as in the example from a 7 month DBA/2J mouse. VEP amplitudes were plotted as a function of spatial frequencies, semilog coordinates, for each eye, i.e. the eye treated with topical applications of BDNF (12 µg/µl; black circles, solid line indicates the linear fit) and the contralateral eye treated with vehicle (empty circles, dashed line indicates the linear fit); cortical spatial resolution limit for each eye (BDNF treated and vehicle treated eye) is indicated by an arrow. **D**. The averaged cortical spatial resolution limit (cortical acuity) in the BDNF treated eyes was significantly higher than that in the vehicle treated eyes; *p<0.05. Error bars indicate SEM.

To complete the visual function assessment we recorded VEPs for stimulation of BDNF and vehicle treated eye (see examples in Fig. 5A). VEP amplitudes at spatial frequencies of 0.05 and 0.2 c/deg (Fig. 5B) were significantly increased in BDNF treated eyes (12 µg/µl BDNF, n mice = 8, eyes = 8; 0.05 c/deg mean amplitude = 11.84 µV, SEM = 0.45 and 0.2 c/deg mean amplitude = 8 µV, SEM = 0.74; contralateral vehicle treated eyes = 8; 0.05 c/deg mean amplitude = 7.6 µV, SEM = 0.2 and 0.2 c/deg mean amplitude = 2.57 µV, SEM = 0.15; p<0.05). Subsequently, we measured the cortical spatial resolution limit, an electrophysiological correlate of the behavioral visual acuity. The cortical spatial resolution limit in each mouse eye was calculated by means of linear extrapolation of data points to the noise level in 7 month DBA/2J mice as in the example reported in Fig. 5 (C). The novel result (Fig. 5D) is that BDNF topical eye treatment was able to rescue the mean cortical spatial resolution limit (n = 8 eyes, mean = 0.53 c/deg, SEM = 0.05) that was drastically reduced in the DBA/2J mice whose eye was treated with vehicle (n = 8 eyes, mean = 0.26 c/deg, SEM = 0.03; p<0.05).

Thus, repeated topical eye treatment with BDNF was able to restore visual responses in DBA/2J mice with ocular hypertension as evaluated by P-ERG and VEP recordings.

**Figure 6:**
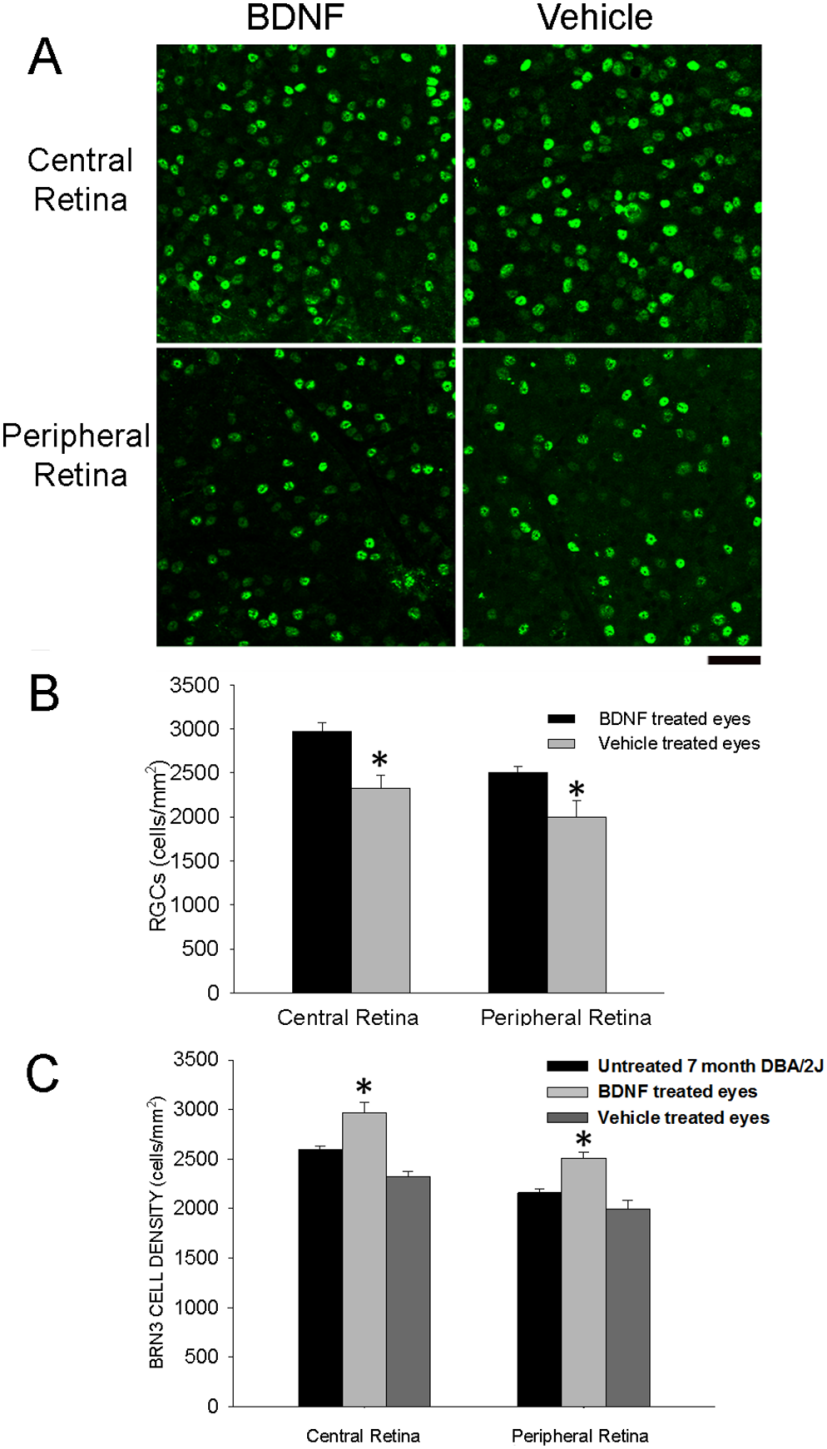
Effects of topical eye treatment with BDNF on RGCs expressing Brn3. Topical eye treatment was performed as already described in Fig. 4 and 5; at the end of the P-ERG recording session mice were sacrificed for immunocytochemistry. **A**. Magnifications from the central (top row) and peripheral (bottom row) field from flat mounted retinas showing Brn3 immunopositive RGCs in BDNF treated eye (left column) and contralateral vehicle treated eye (right column) of a 7 month DBA/2J mouse; scale bar = 40 µm. **B**. All immunopositive RGCs were counted independently from the level of Brn3 expression. The histogram reports the mean cell density of RGCs expressing Brn3 (cells/mm^2^) in the central and peripheral retina from BDNF treated eye (12 µg/µl); Brn3 immunopositive RGC cell density was significantly higher in both the central and peripheral retina of BDNF treated eye respect to vehicle treated eye, *p<0.05 (one-way ANOVA). **C**. Summary of Brn3 results obtained in 7 month DBA/2J untreated eyes, 7 month DBA/2J BDNF treated and vehicle treated eyes; BDNF treated eyes *versus* vehicle treated eyes and untreated eyes, *p<0.05 (one-way ANOVA).

At the end of P-ERG recording session retinal tissue was processed using immunocytochemistry for Brn3. In Fig. 6 (A) we reported images of whole mount retina showing immunolabeled RGCs in the central and peripheral retina of 7 month DBA/2J mice. We quantified the cell density of Brn3 immunolabelled cells in the retinas of BDNF and vehicle treated eye; histograms in Fig. 6 (B) show a significantly higher number of immunolabeled RGCs in the central and peripheral retina of the BDNF treated eye (12 µg/µl BDNF, n mice 9; eyes = 9; mean central retina = 2971, SEM = 96 and mean peripheral retina = 2507, SEM = 65; vehicle, n eyes = 9; mean central retina = 2335.43, SEM = 49.72 and mean peripheral retina = 2007.16, SEM = 78.88; BDNF treated eyes *vs* vehicle treated eyes, p<0.05). In the panel C of Fig. 6 we reported a summary histogram showing the cell density of RGCs expressing Brn3 (central and peripheral retina) in 7 month DBA/2J untreated eyes, BDNF treated and vehicle treated eyes of 7 month DBA/2J mice.

Altogether topical eye repeated applications showed that BDNF treatment was able to restore visual responses and, at least partially, RGCs Brn3 expression in DBA/2J mice with elevated IOP.

### Topical eye treatment with BDNF increases BDNF retinal level

**Figure 7:**
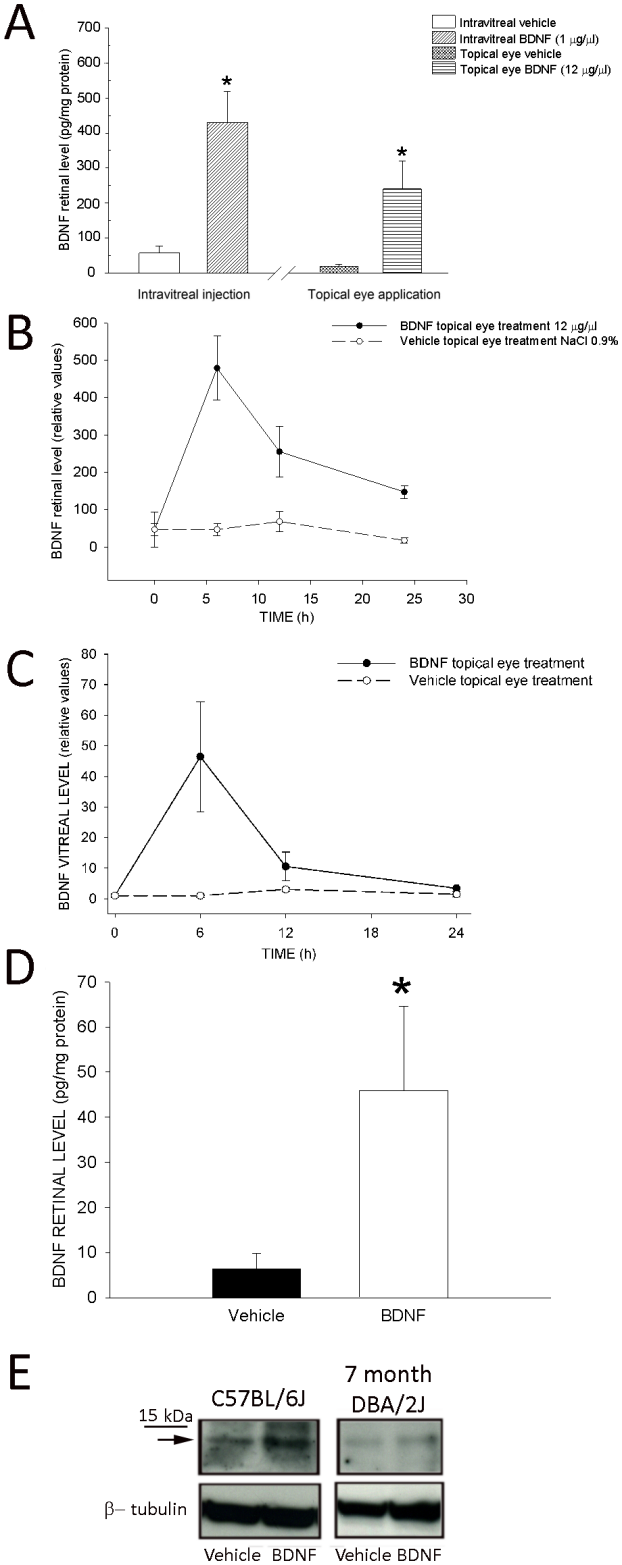
Retinal level of BDNF following intravitreal and topical eye treatment in rat and mouse. **A**. The left part of the histogram shows the rat retinal BDNF level (pg/mg protein, ELISA) in the BDNF injected eye (BDNF concentration = 1 µg/µl in physiological solution, volume 2 µl; n = 6 retinas) and vehicle injected eye (physiological solution, volume = 2 µl; n = 6 retinas). The right part of the figure reports the rat retinal level of BDNF following single topical eye treatment with BDNF (concentration = 12 µg/µl, volume = 10 µl, n = 5 retinas) respect to vehicle treated eye (physiological solution, volume = 10 µl). Retinal level of BDNF was determined by ELISA 6 h after treatment in Wistar rats; * p<0.05 (Student’s *t* test). **B, C**. Pharmacokinetic of BDNF topical eye application in the rats assessed using ELISA. Topical eye treatment with BDNF (concentration of BDNF = 12 µg/µl, volume = 10 µl of NaCl 0.9%) showed that the mean BDNF relative level in the retina (B) and vitreous humor (C) of eyes (n = 5) was higher when measured 6 h after treatment; 12 h after treatment, the level of BDNF in the retina and vitreous humor (n = 4) was still increased. Retinal values from BDNF treated eyes were normalized with respect to those measured in the contralateral eyes before treatment (time 0 in the panel) and averaged. **D**. BDNF retinal level in C57BL/6J mice assessed using ELISA. BDNF treated eyes (n = 5; concentration of BDNF = 12 µg/µl, volume = 5 µl of NaCl 0.9%) show higher BDNF retinal level with respect to vehicle treated eyes; *p<0.05. Vertical bars indicate SEM. **E**. Western blot in C57BL/6J mouse (left panel) from the retina of eye treated with BDNF (concentration of BDNF = 12 µg/µl, volume = 5 µl of NaCl 0.9%, right column) and vehicle, in 7 month DBA/2J mice with ocular hypertension (right panel) from the retina of eye treated with BDNF (concentration of BDNF = 12 µg/µl, volume = 5 µl of NaCl 0.9%; right column) and vehicle. The arrow indicates the BDNF band, together with a marker of m.w. (15 KDa); β-tubulin is reported at the bottom of each panel. We used anti-BDNF antibody AB1534SP (see Material and Methods).

To estimate permeability of BDNF into the posterior eye segment we explored whether single topical eye application of BDNF at the level of conjunctival fornix resulted in the increase of retinal BDNF. To this aim we measured BDNF level in the retina of rat and mouse; we used two animal species differing by eye volume (the mouse eye is one eighth of a rat eye) to test whether diffusion was limited by size. We compared BDNF retinal level in the rat using intravitreal injection and topical eye treatment (Fig. 7A); for topical eye application we used the same concentration proven to be effective in DBA/2J mouse (12 µg/µl) and a bigger volume of solution (10 µl). Retinal BDNF level was significantly increased respect to the contralateral eye when BDNF was intravitreally injected (Fig. 7A, left side) at the concentration of 1 µg/µl (2 µl of physiological solution; n = 6 retinas, BDNF mean = 429.88 pg/mg protein, SEM = 89; n = 6 retinas, vehicle mean = 56.8 pg/mg protein, SEM = 20; p<0.05) and measured 6 h after by ELISA. Similarly, one single drop (10 µl) of physiological solution containing BDNF at the concentration of 12 µg/µl in the conjunctival sac (Fig. 7A, right side) was able to significantly increase retinal BDNF (n = 5 retinas, BDNF mean = 240 pg/mg protein, SEM = 80; n = 5 retinas, vehicle mean = 19.5 pg/mg protein, SEM = 5; p<0.05). The pharmacokinetic study of BDNF topical eye application showed that BDNF retinal level (Fig. 7B) was higher when measured 6 h after treatment returning slowly towards basal level by 24 h in the rat retina; a similar time course was present when BDNF was measured in the vitreous humor of the eye (Fig. 7C). Importantly, 12 h after treatment, the level of the protein was still elevated over basal value both in the retina and vitreous humor. Also, we showed in the Fig. 7 (D) that topical eye application of BDNF solution (5 µl at the concentration of 12 µg/µl) in the C57BL/6J mouse conjunctival fornix increased the retinal content of BDNF (n eyes = 5; ELISA, mean value = 45.92 pg/mg protein, SEM = 18.74) with respect to vehicle treated eyes (n eyes = 5; mean value = 6.28 pg/mg protein, SEM = 3.5). Since the retinal level in vehicle treated eyes was at the low limit of the Elisa kit we decided to use western blot in addition. In Fig. 7 (E) we reported that following BDNF topical eye treatment (5 µl at BDNF concentration of 12 µg/µl) the retinal level was increased in the BDNF treated retina of C57BL/6J mouse, thus confirming previous results obtained by ELISA; in addition, we reported a slight increase of BDNF retinal level in 7 month DBA/2J mouse with respect to that of vehicle treated eye. Thus, a single drop of BDNF in the conjunctival fornix at the concentration of 12 µg/µl was able to increase the retinal level of BDNF in rat and mouse.

## Discussion

DBA/2J mouse is the most frequently used model of spontaneously arising elevation of intraocular pressure (IOP) and glaucoma. DBA/2J has two mutated genes, tyrosinase-related protein 1 (*Tyrp1*) and glycoprotein non-metastatic melanoma protein B (*Gpnmb*), causing iris disease and IOP elevation that results in progressive RGC degeneration and atrophy of optic nerve [3]. RGCs and optic nerve fibers express the BDNF receptor TrkB in adult retina [21], [22], [23], [32], BDNF is anterogradely and retrogradely transported in optic nerve fibers [33], [34], [35]. BDNF exerts neuroprotection in different animal models of optic nerve injury and diseases. In particular, RGC death induced by axotomy of optic nerve in rodents was rescued by BDNF [18], [20]; administration of BDNF at the time of optic nerve crush preserved the morphology of injured RGCs in cats [25]. Since most of RGCs are trophically dependent on BDNF we think that DBA/2J represents a proper experimental model to test BDNF rescue of visual responses.

We showed that IOP is already elevated in 6 month DBA/2J mice in agreement with previous results reporting IOP increase from an age of 5–6 months [10], [36]. IOP discrepancies (as examples see [10], [36]) and similarities (as examples see [4], [37]) on IOP actual values between our work and previous papers are due to different methodology (for example anaesthetized vs. unanaesthetized mice, mice stressed by IOP measurements vs. trained non stressed mice as in the present work).

There is general agreement that the IOP increase induces functional impairment of inner retina [38] preceding the atrophy of optic nerve and RGC soma loss in mouse models of glaucoma [5], [16], [17]. We used the steady-state P-ERG to monitor progressive impairment of retinal visual function in DBA/2J mice. The bulk of evidence in animal models and patients [6]–[10], [13], [28] showed that P-ERG is an adequate tool with regards to signal-to-noise ratio, reproducibility, and sensitivity to detect the functional changes in the inner retina where its generators were found [11], [12]. P-ERG and VEP were recorded in DBA/2J mice at different ages: 4 months with normal IOP, 6 and 7 months when IOP was already elevated. We showed that visual responses recorded by P-ERG and VEP decrease in amplitude as a function of IOP increase in DBA/2J mice. Since the P-ERG and VEP were recorded with different methods of stimulation and there is a substantial RGC-LGN convergence [39] we cannot eliminate the possibility that neuronal responses in different areas along the post-retinal visual pathways might contribute to VEP impairment. Similarly to P-ERG the cortical responses evaluated by VEPs were unaffected in 4 month DBA/2J mice. Although indirectly, the result that P-ERG and VEP were unaffected in 4 month DBA/2J mice with normal IOP rules out the possibility that impairment of visual responses was due to pleiotropic effects of the two mutated genes in DBA/2J mice.

Early anomalies in RGCs induced by ocular hypertension were evaluated using an antibody that recognizes *Brn3* genes (*a, b, c*) products. In the retina, *Brn3* genes are expressed in RGCs controlling their differentiation, dendritic stratification and axonal development [15]. Xiang et al. [14] reported that 36% of the cells in the adult mouse ganglion cell layer (GCL) were Brn3a immunopositive and 35% were Brn3b immunopositive while Brn3c only labeled 15% of neurons in the GCL. Because in the GCL approximately one half of the neurons are RGCs then, around 60–70% of RGCs in mice express/co-express Brn3a, Brn3b and Brn3c. Previous work showed that antibody recognizing Brn3 can be considered as a good and reliable marker to detect RGCs in healthy and damaged retinas [40], [41] including experimental glaucoma. We found that RGCs expressing Brn3 were reduced in 7 month DBA/2J mice with ocular hypertension. Although we did not quantify the expression of Brn3 in single RGC, we suggest down regulation of Brn3 expression rather than cell loss, also in consideration of the fact that using methods to detect apoptotic RGCs we reported negative results in our experimental context; indeed, there is a general agreement that RGC soma loss becomes detectable when DBA/2J mice aged 11–15 months [5], [16], [17]. In agreement with this idea we reported that cell death assessed using the Tunel method was absent in 7 month DBA/2J mice with ocular hypertension. Furthermore, in S1 Fig. we showed that γ-Synuclein, a protein highly expressed in retinal ganglion cells (RGCs), frequently used to assess RGC cell viability [42], was unaffected in 7 month DBA/2J mice starting to be reduced at a later age (11–14 months).

We did not investigate whether specific subtypes of RGCs expressing Brn3 were especially affected in 7 month DBA/2J mice. Brn3 altered expression was related to IOP rise in DBA/2J mice, similarly to what was shown for P-ERG and VEP. Thus, RGCs Brn3 expression might be considered as an early marker of RGCs dysfunction related to IOP rise.

We showed that BDNF supplied by a few intravitreal injections was able to preserve P-ERG in 7 month DBA/2J mice with ocular hypertension in agreement with previous results [26]; however, multiple intravitreal injections are unlikely to be a sustainable treatment for an eye affected by ocular hypertension. For this reason we focused on topical treatment with BDNF eye-drops. P-ERG and VEP amplitudes were the first electrophysiological parameters improved in 7 month DBA/2J mice whose eyes were repeatedly treated with BDNF drops in the conjunctival fornix. Also, distressed RGCs expressing Brn3 were partially rescued by BDNF topical eye treatment.

Thus, we reported that repeated topical BDNF eye applications at the level of conjunctival fornix was able to rescue visual retinal and cortical responses in DBA/2J mice with elevated IOP. We showed that BDNF effects were independent of IOP reduction.

To know whether the restoration of P-ERG and VEP responses may correspond to an improvement of the visual function we looked at the BDNF impact on visual cortical acuity. VEPs were previously used to assess the spatial resolution limit (cortical acuity) in mice [28], [43]. The mouse spatial resolution limit is in the order of 0.5–0.55 c/deg as assessed by an operant visual behavioral task [28], [44] and 0.6–0.65 c/deg if VEP recordings are used [28]. Remarkably, the visual cortical acuity was largely preserved by BDNF topical eye treatment in 7 month DBA/2J mice.

Rescue of retinal visual responses by BDNF is in line with the presence of the BDNF receptor TrkB in RGCs and optic nerve fibers of adult mammals, anterograde/retrograde axonal transport of TrkB and BDNF by RGC axons. Interestingly, acute glaucoma in rats induces an increase of TrkB in optic nerve fibers and TrkB was identified in axons of monkey optic nerve heads with glaucoma [45]. In rodent experimental models of glaucoma with elevated IOP the retinal levels of either BDNF or its receptor were unaffected [46], [47]. Recent evidence reported the interruption of BDNF retrograde transport and accumulation of TrkB at the optic nerve head in acute and chronic glaucoma models suggesting a role for BDNF retrograde signaling in RGC degeneration in glaucoma [5], [45] and RGC normal function [48].

Pharmacologically active molecules can reach the retina through two routes when topically applied to the eye: through the cornea and through the conjunctiva. Since BDNF is high molecular weight protein, it is very unlikely that it can reach the retina by crossing the cornea. In a previous report, the NGF was topically applied at the level of conjunctival fornix. By measuring the NGF level in the conjunctiva and/or sclera the authors suggested that NGF reached the posterior eye segment travelling through the conjunctiva/sclera [49]. Trans-conjunctiva/scleral delivery requires BDNF to permeate through several layers of ocular tissue (conjunctive/sclera, Bruch’s membrane-choroid, retinal pigment epithelium) to reach the retina. As a result of these barriers, a steep concentration drop of BDNF from the conjunctive/sclera to the retina is expected and therefore a high concentration/amount of BDNF on the ocular surface should be used for retinal delivery. Since retinal vessels, in particular vascular endothelium and associated cells, express BDNF receptors [50], [51] there is the possibility that part of supplied BDNF might be captured and eliminated by the retinal vasculature. By comparing intravitreal injection with topical eye treatment, we showed that the effective dose inducing the increase of the retinal BDNF level is higher for topical eye treatment with BDNF, in respect to intravitreal injection. Beyond these considerations we showed that BDNF level in the retina of C57BL/6J and 7 month DBA/2J mice was increased following topical eye BDNF application. Potential toxic effects on the cornea and/or conjunctive following topical eye application should be taken into account, especially when the pH of the solution is too low and the solution osmolarity out of the physiological range; future research on BDNF vehicles will be essential to increase the BDNF penetrability and eye safety following topical eye application.

## Conclusion

Topical eye treatment with BDNF can be pursued to elevate the BDNF retinal level leading to rescue of visual retinal responses in DBA/2J mice during an early stage of retinal degeneration characterized by ocular hypertension although a high concentration/amount of this neurotrophin should be used. It has to be proved whether protracted treatment with BDNF instead of short treatment as reported here will be feasible for the rodent and, possibly, human retina to prevent RG cell death and optic nerve atrophy in glaucoma. In addition, it has to be clarified whether BDNF treatment might result in long term efficacy, therefore, it contributes to answering to the question about how long BDNF topical treatment maintains its potency.

## Supporting Information

S1 Fig **RGCs expressing γ-Synuclein in DBA/2J mice.** Retinal ganglion cells (RGCs) labeled by an antibody recognizing γ-Synuclein (A), a member of small unfolded proteins (Synucleins), used to assess RGCs viability appear unaffected in 7 month DBA/2J (bottom panels); top panels report RGCs labeled in C57BL/6J mice. Scale bar = 40 µm. The histogram reported in B shows the cell density of γ-Synuclein immunopositive RGCs (see Material and Methods for cell sampling in the whole mount retina) in C57BL/6J mice and in DBA/2J mice. Gamma-Synuclein immunopositive RGC density in 7 (n mice = 4, 8 eyes; central retina mean cell density = 7997, SEM = 240; peripheral retina mean cell density = 7210, SEM = 217) and 8 month DBA/2J mice (n mice = 3, 6 eyes; central retina mean cell density = 7674.67, SEM = 235.29; peripheral retina mean cell density = 6772, SEM = 235.4); γ-Synuclein immunopositive RGCs in 7 and 8 month DBA/2J mice were not significantly different with respect to C57BL/6J (n mice = 4, 8 eyes; central retina mean cell density = 7876, SEM = 456; peripheral retina mean cell density = 6621, SEM = 325) and 4 month DBA/2J mice (n mice = 4, 7 eyes; central retina mean cell density = 7674, SEM = 235; peripheral retina mean cell density = 6772, SEM = 230). At the age of 11 months RGCs expressing γ-Synuclein were significantly reduced in the peripheral retina (n mice = 3, 6 eyes; mean in the central retina = 7451.87, SEM = 136.39; mean in the peripheral retina = 5892.74, SEM = 232.35) while at later ages both the central and peripheral retina resulted affected (14 months of age n mice = 3, eyes 6, mean cell density in central retina = 4866, SEM = 261.27; mean cell density in peripheral retina = 4682, SEM = 366.13; 17 months, n mice = 3, eyes = 5; mean cell density in central retina = 3634.66, SEM = 632.77; mean cell density in peripheral retina = 3632, SEM = 287.21). Different groups of DBA/2J mice were compared with C57BL/6J mice; *p<0.05 (one-way ANOVA). Error bars indicate SEM. From the results it is clear that γ-Synuclein immunopositive RGCs did not decrease in 7 month DBA/2J mice with respect to C57BL/6J and 4 month DBA/2J mice.
(TIF)
