# Supplementary figures and images for: Rescue of Retinal Function by BDNF in a Mouse Model of Glaucoma
Source: PLoS One. 2014 Dec 23;9(12):e115579. doi: 10.1371/journal.pone.0115579 (PMC4275209; doi:10.1371/journal.pone.0115579)

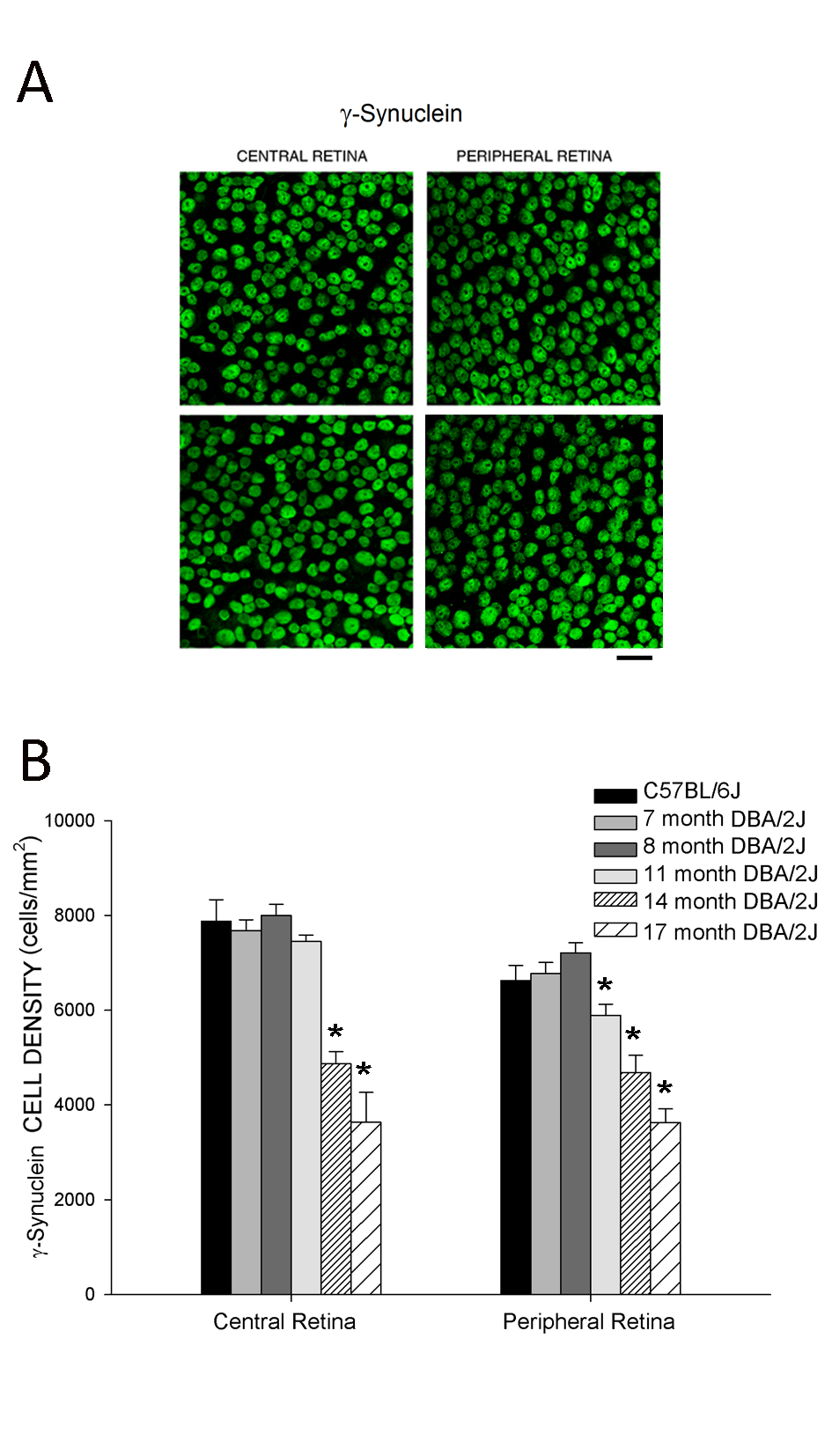

Supplement: S1 Fig — RGCs expressing γ-Synuclein in DBA/2J mice. Retinal ganglion cells (RGCs) labeled by an antibody recognizing γ-Synuclein (A), a member of small unfolded proteins (Synucleins), used to assess RGCs viability appear unaffected in 7 month DBA/2J (bottom panels); top panels report RGCs labeled in C57BL/6J mice. Scale bar = 40 µm. The histogram reported in B shows the cell density of γ-Synuclein immunopositive RGCs (see Material and Methods for cell sampling in the whole mount retina) in C57BL/6J mice and in DBA/2J mice. Gamma-Synuclein immunopositive RGC density in 7 (n mice = 4, 8 eyes; central retina mean cell density = 7997, SEM = 240; peripheral retina mean cell density = 7210, SEM = 217) and 8 month DBA/2J mice (n mice = 3, 6 eyes; central retina mean cell density = 7674.67, SEM = 235.29; peripheral retina mean cell density = 6772, SEM = 235.4); γ-Synuclein immunopositive RGCs in 7 and 8 month DBA/2J mice were not significantly different with respect to C57BL/6J (n mice = 4, 8 eyes; central retina mean cell density = 7876, SEM = 456; peripheral retina mean cell density = 6621, SEM = 325) and 4 month DBA/2J mice (n mice = 4, 7 eyes; central retina mean cell density = 7674, SEM = 235; peripheral retina mean cell density = 6772, SEM = 230). At the age of 11 months RGCs expressing γ-Synuclein were significantly reduced in the peripheral retina (n mice = 3, 6 eyes; mean in the central retina = 7451.87, SEM = 136.39; mean in the peripheral retina = 5892.74, SEM = 232.35) while at later ages both the central and peripheral retina resulted affected (14 months of age n mice = 3, eyes 6, mean cell density in central retina = 4866, SEM = 261.27; mean cell density in peripheral retina = 4682, SEM = 366.13; 17 months, n mice = 3, eyes = 5; mean cell density in central retina = 3634.66, SEM = 632.77; mean cell density in peripheral retina = 3632, SEM = 287.21). Different groups of DBA/2J mice were compared with C57BL/6J mice; *p<0.05 (one-way ANOVA). Error bars indicate SEM. From [file pone.0115579.s001.tif]
